# *NR5A1* gene variants repress the ovarian‐specific WNT signaling pathway in 46,XX disorders of sex development patients

**DOI:** 10.1002/humu.23672

**Published:** 2018-11-30

**Authors:** Ingrid M. Knarston, Gorjana Robevska, Jocelyn A van den Bergen, Stefanie Eggers, Brittany Croft, Jason Yates, Remko Hersmus, Leendert H. J. Looijenga, Fergus J. Cameron, Klaus Monhike, Katie L. Ayers, Andrew H. Sinclair

**Affiliations:** ^1^ Murdoch Children's Research Institute Melbourne Australia; ^2^ Department of Paediatrics The University of Melbourne Melbourne Australia; ^3^ The Hudson Institute of Medical Research Monash University Melbourne Australia; ^4^ The Townsville Hospital Department of Health Queensland Australia; ^5^ Department of Pathology Josephine Nefkens Building Erasmus University Medical Centre Rotterdam The Netherlands; ^6^ Department of Endocrinology and Diabetes Royal Children's Hospital Melbourne Australia; ^7^ Otto‐von‐Guericke Universität Universitätskinderklinik Magdeburg Germany

**Keywords:** 46,XX ovotesticular DSD, 46,XX testicular DSD, disorders of sex development, NR5A1, SF1

## Abstract

Several recent reports have described a missense variant in the gene *NR5A1* (c.274C>T; p.Arg92Trp) in a significant number of 46,XX ovotesticular or testicular disorders of sex development (DSDs) cases. The affected residue falls within the DNA‐binding domain of the NR5A1 protein, however the exact mechanism by which it causes testicular development in 46,XX individuals remains unclear. We have screened a cohort of 26 patients with 46,XX (ovo)testicular DSD and identified three unrelated individuals with this *NR5A1* variant (p.Arg92Trp), as well as one patient with a novel *NR5A1* variant (c.779C>T; p.Ala260Val). We examined the functional effect of these changes, finding that while protein levels and localization were unaffected, variant NR5A1 proteins repress the WNT signaling pathway and have less ability to upregulate the anti‐testis gene *NR0B1*. These findings highlight how *NR5A1* variants impact ovarian differentiation across multiple pathways, resulting in a switch from ovarian to testis development in genetic females.

## 1 INTRODUCTION

46,XX (ovo)testicular differences/disorders of sex development (DSDs; MIM# 617480) are a rare group of phenotypes where the testicular differentiation pathway is activated during development in a 46,XX individual, resulting in the formation of testes (46,XX testicular DSD) or ovotestes (46,XX ovotesticular DSD). Broadly, these conditions are caused by gain‐of‐function variants in genes important for testis differentiation or loss‐of‐function variants in ovarian differentiation genes (reviewed in Knarston, Ayers, & Sinclair, 2016). Up to 90% of 46,XX testicular DSDs (McElreavey, Vilain, & Abbas, 1993) and 10% of 46,XX ovotesticular DSDs (Vilain et al., 2011) are caused by translocation of the *Sex‐determining region Y* (*SRY*) gene, the initiating gene for testis differentiation, onto the X chromosome (*SRY*‐positive 46,XX DSD). Another common cause is ectopic *SRY‐box 9* (*SOX9*) expression, often caused by duplications in the upstream enhancer (Croft, Ohnesorg, & Sinclair, 2018). Loss of function variants in ovarian pathway genes (e.g., *R‐Spondin 1* [*RSPO1*]) have been identified in a small subset of syndromic 46,XX (ovo)testicular DSDs (Bashamboo et al., 2018; Parma et al., 2006; Tomaselli et al., 2008). Despite these known causes, SRY‐negative 46,XX (ovo)testicular DSDs have a diagnostic rate much lower than that of other DSDs (Eggers et al., 2016) highlighting a need to identify novel genes or genetic variants underlying these DSDs. Recently, five reports have described a recurrent heterozygous variant in the *Nuclear Receptor Subfamily 5 Group A Member 1* (*NR5A1*) gene (c.274C>T; p.Arg92Trp) in 12 cases of 46,XX (ovo)testicular DSD (Baetens et al., 2017; Bashamboo et al., 2016; Domenice et al., 2016; Igarashi et al., 2017; Takasawa et al., 2017), although the underlying mechanism for how this *NR5A1* variant causes sex reversal in these cases is still unclear. NR5A1, or steroidogenic factor‐1 (SF1, Ad4BP; MIM# 184757), is a nuclear receptor transcription factor with a key regulatory role in development of the hypothalamus–pituitary–gonadal and hypothalamus–pituitary–adrenal axes (Morohashi et al., 1996; Parker et al., 1997; Val, Lefrancois‐Martinez, Veyssiere, & Martinez, 2003), and thus in the development of the male and female gonads (reviewed in Ferraz‐de‐Souza, Lin, & Achermann, 2011; Lin et al., 2008). Human variations in *NR5A1* have been widely characterized in 46,XY DSDs (gonadal dysgenesis, hypospadias, undervirilization, and male infertility; Robevska et al., 2018; Ropke et al., 2013) and 46,XX primary ovarian insufficiency (Voican et al., 2013) and commonly show variable phenotypic expressivity and incomplete penetrance. In a recent study, we applied massively parallel sequencing of a targeted gene panel to a DSD cohort, which included 26 patients with 46,XX (ovo)testicular DSD (Eggers et al., 2016). From this study, we identified three new cases of 46,XX (ovo)testicular DSD with the *NR5A1* c.274C>T; p.Arg92Trp variant. Furthermore, we report an additional *NR5A1* variant, c.779C>T; p.Ala260Val, in a case of 46,XX ovotesticular DSD. To our knowledge, this is the first instance of an *NR5A1* variant that does not affect codon 92 occurring in an individual with 46,XX (ovo)testicular DSD. We have used protein modeling, protein localization, and luciferase assays to propose mechanistic insights for *NR5A1*‐mediated XX sex reversal in these cases.

## 2 METHODS

### 2.1 Ethical approval and patient recruitment

Patient recruitment, consent, and DNA extraction were carried out as described previously (Eggers et al., 2016). Ethical approval for this study was obtained from the Human Ethics Committee of the Faculty of Medicine at the Royal Children's Hospital, Melbourne, Victoria, Australia (HREC22073).

### 2.2 Massively parallel sequencing

Total genomic DNA was sequenced on a targeted panel (HaloPlex, Agilent, Santa Clara, CA) that includes 64 diagnostic DSD genes (described in Eggers et al., 2016). This included six genes that have been implicated in 46,XX (ovo)testicular DSD (*FGF9*, *RSPO1*, *SOX3*, *SOX9*, *SOX10*, and *WNT4*). *NR5A1* variant numbering is based on GenBank reference DNA sequence NM_004959.4, with the A of the ATG initiation codon designated +1. NR5A1‐predicted protein annotations are based on NP_004950. Analysis for genomic modifiers was performed by filtering variants using a list of 116 NR5A1‐related genes alongside our previously reported filtering criteria. The initial gene list (*N* = 576) was compiled from data in STRING (https://string-db.org/) and NR5A1 overexpression/knockdown assays (Doghman, Figueiredo, Volante, Papotti, & Lalli, 2013), 116 of these genes were covered by our targeted gene panel (*N* = 1,024).

### 2.3 *In silico* protein structure analysis

HOPE analysis was used to analyze the structural and functional consequences of the variants identified (https://www.cmbi.ru.nl/hope/; Venselaar, Beek, Kuipers, Hekkelman, & Vriend, 2010). We generated predictions of NR5A1 variant protein structure *in silico* using I‐TASSER, an online protein modeling server (https://zhanglab.ccmb.med.umich.edu/ITASSER/; Roy, Kucukural, & Zhang, 2010; Zhang et al., 2008). Predicted crystal structures were visualized using PyMOL Molecular Graphics System v1.7.6.6 Enhanced for Mac OS X (https://www.pymol.org).

### 2.4 Protein immunofluorescence

COS‐7 cells were seeded onto eight‐well chamber slides (Lab‐Tek; Brendale, Queensland, Australia) and transfected with NR5A1 expression vectors (WT, p.Arg92Trp, p.Ala260Val) using Lipofectamine 2000 (Invitrogen, Carlsbad, CA). Following 24 hours of transfection, cells were treated and analyzed as described previously (Robevska et al., 2018).

### 2.5 Plasmid construction

Mutant NR5A1 expression vectors containing the p.Arg92Trp or p.Ala260Val variants were created by site‐directed mutagenesis (QuikChange II XL Site‐directed Mutagenesis Kit; Agilent Technologies Inc., Santa Clara, CA) according to the manufacturer's instructions (see primer sequences in Supporting Information Table S1) using the mammalian expression vector pCMV6‐Entry‐*hNR5A1* (RC207577; OriGene Technologies Inc., Rockville, MD) as a template. The pGL4‐*hDAX1* reporter plasmid was generated via cloning of 994 bp of the promoter region of the human *NR0B1* gene (chrX:30327432–30328425) into the pGL4.10.luc2 reporter plasmid (see primer sequences in Supporting Information Table S1).

### 2.6 Luciferase assays

Luciferase reporter assays were performed in two cell lines (HEK 293‐T or COS‐7) in 96‐well plates using Lipofectamine 2000 and the Dual‐Luciferase Reporter 1000 Assay System Kit (Promega; Fitchburg, WI), with co‐transfection of *Renilla* (pRL‐TK) as a marker of transfection efficiency. To assay the NR5A1‐mediated transactivation of *mTESCO*, COS‐7 cells were co‐transfected with a reporter construct, pGL4‐mTESCO (75 ng/well), p*RL‐TK* (10 ng/well), SOX9 (50 ng/well), NR5A1 (WT, p.Arg92Trp, p.Ala260Val and p.Arg84His which was used as a control; 50 ng/well), and FOXL2 (50 ng/well); cells were harvested 24 hours post transfection. The *mTESCO* reporter was used to assay NR0B1‐mediated repression of SOX9, as previously described (Igarashi et al., 2017). Interaction of NR5A1 with *NR0B1* was assayed in HEK 293‐T cells using a pGL4‐hDAX1 reporter as previously described (Mizusaki et al., 2003). To assay the ability of NR5A1 proteins to regulate the canonical WNT pathway, we used the TOPFlash‐TCF reporter as previously described (Bashamboo et al., 2016). Luciferase activity was measured on an Infinite M200 Pro plate reader (Tecan; Männedorf, Zürich, Switzerland); data represent the mean with standard error of three independent experiments, each performed in triplicate.

## 3 RESULTS

### 3.1 Clinical phenotype

All of the individuals presented have a 46,XX karyotype (*SRY*‐negative), with varying degrees of virilization (Table 1). Patient 1 was born to nonconsanguineous parents and recruited as an adult male with azoospermia and bilateral testes; he had undergone an orchidopexy in childhood. Patient 2 presented at birth with ambiguous genitalia, a phallus measuring 2 cm, perineal urethral opening, and bilaterally palpable gonads. Histological evaluation showed them to be bilateral ovotestes. There were no Müllerian structures detected, and testosterone production declined over time. The child underwent orchidopexy and was raised as a female. Patient 3 presented with virilization and was raised as a male; he had a micropenis (stretched penile length of 3 cm at 5 years), hypospadias, and small underdeveloped scrotum with no evidence of Müllerian structures and a positive testosterone response to stimulation. This individual also has a seizure disorder managed by neurologists. Patient 4 was initially raised as a male, then as female from 4 years of age. The patient presented with ambiguous genitalia, a small phallus and vagina (4 cm deep), with separate urethral and vaginal openings. The right gonad was an ovotestis with sporadic germ cells in the tubules, calcifications, and primordial follicles in ovarian part. The left gonad was ovarian tissue with both primordial and developing follicles. Patients 1–3 were found to have normal adrenal function based on hormonal profiling at the time of sample collection, these data were not available for Patient 4.

**Table 1:** Clinical, anatomical, and biochemical characteristics in four 46,XX (ovo)testicular DSD cases

Patient ID	Karyotype	Sex of rearing	External genitalia	Gonadal location (R/L)	Gonadal histology (R/L)	Müllerian structures	Basal gonadotropins	Basal testosterone	T resp. hCG	Adrenal function	AMH	Additional Information
1	46,XX	Male	Microphallus, penile hypospadias	Small testes both 1 ml by orchidometer, right in neck of scrotum left scrotal	Bilateral testes	Not found	High LH and FSH	Normal	ND	Normal	ND	Cardiac murmur, azoospermic
2	46,XX	Female	Microphallus, perineal urethral opening	Bilateral palpable in inguinal region	Bilateral ovotestes	None	ND	2 days old: high T (5.3 nmol/L) 6 y old: basal: low (<0.5)	1 y 7 m old after HCG: Normal (9.3) 6 y old: after HCG: poor response (>1.8)	Normal	ND	Intratubular neoplasia
3	46,XX	Male	Microphallus, hypospadias, underdeveloped scrotum	Bilateral scrotal	ND	None	Low at 7 y old—age appropriate	Low (<0.3 nmol/L [NR: <0.5])	3 m old—normal (6.5 nmol/L [NR: 4–12])	Normal	ND	Seizure disorder
4	46,XX	Initially male, from 4y female	Microphallus, urethra opening and vaginal opening	Both abdominal	R ovotestis, L ovary	Uterus and fallopian tube on both sides	Low but done at age 2 y old	Low but done at age 2 y	Not available	ND	Not available before gonadectomy	

AMH, anti Müllerian hormone; d, days; FSH, follicle stimulating hormone; hCG, Human chorionic gonadotropin stimulation test; LH, luteinizing hormone; m, months; ND, not done; NR, normal range; T, testosterone; y, years.

### 3.2 Identification of novel and known variants in *NR5A1* associated with 46,XX (ovo)testicular DSD

46,XX (ovo)testicular DSD cases were screened using two assays. Patient DNA was run on our custom multiplex ligation‐dependent probe amplification (MLPA) assay (previously described in Ohnesorg et al., 2017), which screens 10 genes implicated in DSD for copy number variants, including four probes in the enhancer region of *SOX9*. The MLPA showed that Patients 1–4 are negative for common copy number variants in known DSD genes. We then performed massively parallel sequencing on a targeted DSD gene panel (previously described in Eggers et al., 2016) to screen the patients for single nucleotide variants and small insertions/deletions in 64 diagnostic DSD genes. A previously reported variant in exon 4 of *NR5A1* (NM_004959.4:c.274C>T, p.Arg92Trp; Baetens et al., 2017; Bashamboo et al., 2016; Igarashi et al., 2017) was identified in three cases of 46,XX (ovo)testicular DSD (Table 2; Patients 1–3). The change was found to be maternally inherited in Patient 3. The mother was subfertile, having 4–6 menses per year and had difficulty falling pregnant. Interestingly, the maternal aunt and uncle of Patient 3 also experienced fertility issues. A further variant (NM_004959.4:c.779C>T, p.Ala260Val) was found in exon 4 of *NR5A1* in a single case of 46,XX ovotesticular DSD (Patient 4). The NR5A1 p.Ala260Val variant was extremely rare in the population (gnomAD frequency: 4.13e‐6) and predicted to be damaging in two of the four *in silico* algorithms used. It has been previously implicated in 46,XY DSD (Chan et al., 2015). These two *NR5A1* variants were found in heterozygous form and flagged for functional validation.

**Table 2:** *NR5A1* variants identified in 46,XX (ovo)testicular DSD cases

Patient ID	Gene	DNA change	Protein change	Zygosity	Inheritance	Previous publications/reports	*In silico*
1	*NR5A1*	c.274C>T	p.Arg92Trp	Heterozygous	N/A	Baetens et al., 2017; Bashamboo et al., 2016; Igarashi et al., 2017	Damaging
2	*NR5A1*	c.274C>T	p.Arg92Trp	Heterozygous	N/A	Baetens et al., 2017; Bashamboo et al., 2016; Igarashi et al., 2017	Damaging
3	*NR5A1*	c.274C>T	p.Arg92Trp	Heterozygous	Maternal	Baetens et al., 2017; Bashamboo et al., 2016; Igarashi et al., 2017	Damaging
4	*NR5A1*	c.779C>T	p.Ala260Val	Heterozygous	N/A	Chan et al., 2015 gnomAD frequency: 4.13 × 10^–6^	Two of 4 but previously found to be damaging.

*In silico*: PolyPhen2, MutationTaster, SIFT, LRT; Damaging: deleterious or possibly deleterious in 4/4 predictors; N/A, not available. DNA mutation numbering is based on GenBank reference DNA sequence NM_04959.4, with the A of the ATG initiation codon designated +1. Predicted protein annotations are based on NP_004950.

### 3.3 NR5A1 variants have unaffected protein expression and subcellular localization

**Figure 1:**
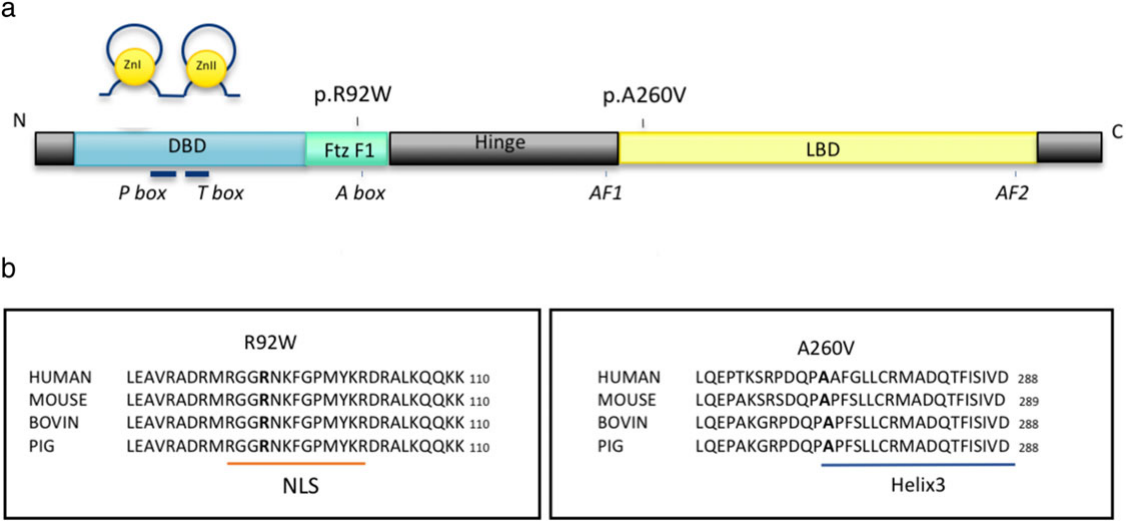
Variants in NR5A1 identified in a cohort of individuals with 46,XX DSD. (a) Schematic representation of the predicted protein structure of NR5A1 showing the approximate location of the two variants identified in a cohort of individuals with 46,XX DSD. The protein domains are as follows: DNA binding domain (DBD) containing two zinc finger motifs (Zn) and the Fushi‐tarazu factor 1 box (Ftz‐F1), the hinge region, and ligand binding domain (LBD). P‐Box, T‐box, A‐box, as well as two activational domains—AF1 and AF2. (b) Evolutionary conservation of the NR5A1 protein sequence around the two missense variants identified in our cohort

**Figure 2:**
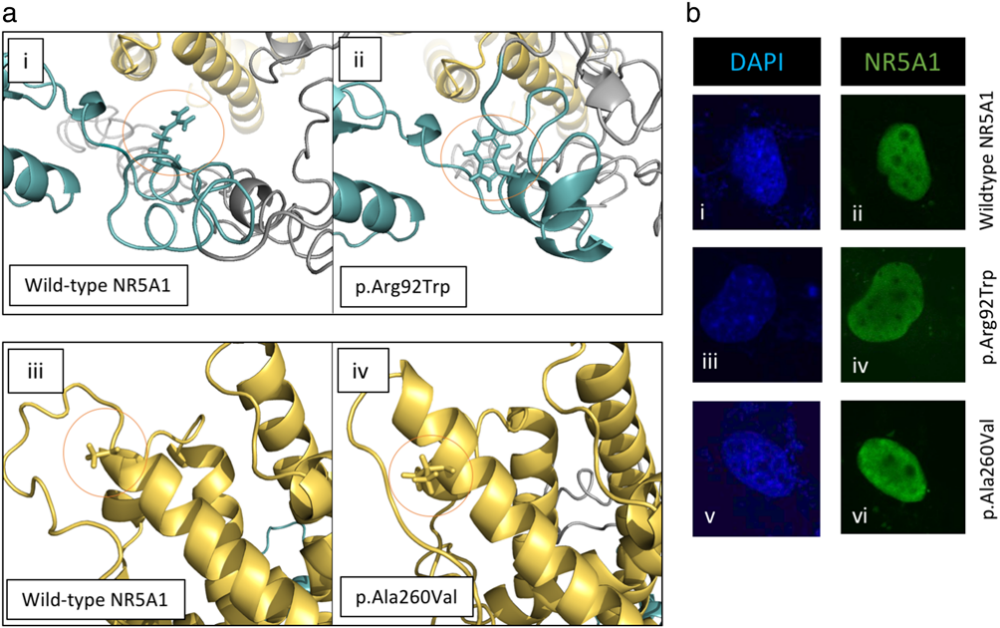
Protein conformation and cellular expression. (a) To investigate the potential effect of the variants on protein conformation, we performed an *in silico* prediction with the wild‐type NR5A1 and both variants using I‐Tasser and PyMol modeling. (a: i and ii) Wild‐type arginine (Arg, R) at position 92 falls within the DNA binding domain of the protein. The mutant tryptophan (Trp, W) is larger than the arginine and has less hydrogen bonding potential. (a: iii and iv) The residue at position 260 falls within alpha helix 3 of the ligand binding domain. The wild‐type alanine (Ala, A) is smaller than the mutant valine (val, V), this is located on the protein surface. (b) Protein expression of both variant and wild‐type NR5A1 was assessed in COS‐7 cells with an NR5A1 antibody (green). Cells were transfected with an equal amount of NR5A1 expression vector (wild‐type or variant). Nuclear counterstaining was performed with DAPI (blue). Wild‐type NR5A1 showed strong nuclear staining with nucleolar exclusions (b: i and ii). The variant NR5A1 protein expression and localization was unaffected (b: iii–vi)

To assess whether the NR5A1 variants affected protein localization or expression, we used immunofluorescence staining (Figure 2b). The wild‐type and p.Arg92Trp variant NR5A1 protein showed strong nuclear expression with nucleolar exclusions (Figure 2b: ii and iv), consistent with previous reports (Baetens et al., 2017; Kohler et al., 2008). We showed that the p.Ala260Val variant NR5A1 protein exhibits similar levels of expression and nuclear localization (Figure 2b: vi). To elucidate how the amino acid substitutions may affect function, we undertook protein structure modeling. At codon 92 of NR5A1, the wild‐type arginine falls within the highly conserved and functionally critical DNA binding domain (Figure 1a,b). As described previously, the substitution of an arginine to tryptophan at position 92 leads to a change in charge (from positive to neutral) as well as a loss of hydrogen bond formation potential (due to size difference between the residues; Figure 2a: i and ii; Baetens et al., 2017; Bashamboo et al., 2016; Domenice et al., 2016; Igarashi et al., 2017). Together these may affect the protein's ability to interact with DNA. At codon 260 of NR5A1, the wild‐type alanine falls within the evolutionary conserved alpha helix 3, part of the ligand binding domain (Figure 2a: iii). This alanine residue is predicted to be on the surface of the protein and replacement with a larger valine may affect interactions between NR5A1 and other molecules (Figure 2a: iv).

### 3.4 NR5A1 variants show decreased activation of male pathway

**Figure 3:**
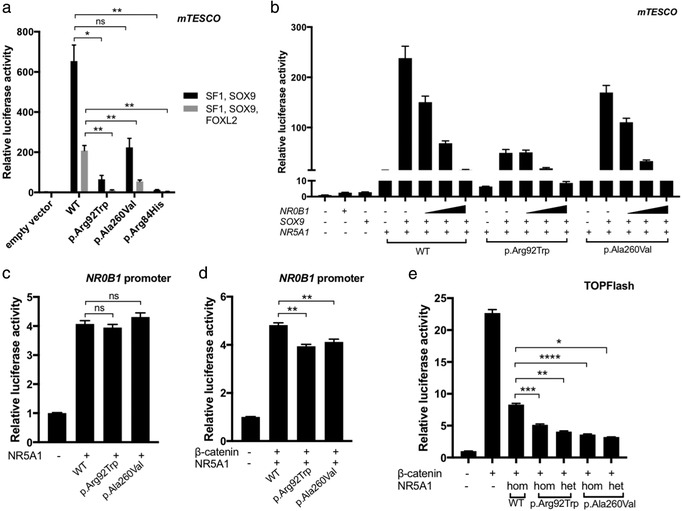
NR5A1 mutants show altered function in luciferase assays using sex differentiation‐specific reporters. (a) Both NR5A1 variants as well as a positive control variant (loss of function from 46,XY DSD) show decreased transactivation of *SOX9 mTESCO* when co‐transfected into COS‐7 cells with SOX9. This is also observed when the female pathway SOX9 repressor, FOXL2, is also transfected. (b) Co‐transfection of HEK 293‐T cells with NR5A1, SOX9, and increasing concentrations of NR0B1 showed that mutant NR5A1 does not affect NR0B1‐mediated repression of SOX9. SOX9 activity was measured using the TESCO reporter. (c) Co‐transfection of COS‐7 cells with wild‐type or mutant NR5A1 shows no change in activity of the NR0B1 promoter for both NR5A1 mutants (d) Co‐transfection of COS‐7 cells with wild‐type or mutant NR5A1 and β‐catenin results in repression of the NR0B1 promoter for both NR5A1 mutants. (e) TOPFlash activation is reduced when HEK 293‐T cells are transfected with β‐catenin and mutant NR5A1 compared to wild‐type NR5A1. Data represent the mean with the standard error of three independent experiments performed in triplicate. An unpaired *t*‐test was applied to obtain *P*‐values, ****P < 0.0001; ***P < 0.001; **P < 0.01; *P < 0.05; ns = *P* > 0.05

It has been previously shown that the NR5A1 p.Arg92Trp variant has reduced ability to upregulate male pathway genes (*Sox9*, *Anti Mullerian hormone* [*Amh*], *Cytochrome P450 Family 11 Subfamily A Member 1* [*Cyp11a1*]; Bashamboo et al., 2016). We wondered if our new variant may show an increased ability to upregulate the testis pathway. To address this, we tested the two NR5A1 variants on their ability to upregulate the *Sox9* enhancer *mTESCO*, as well as a previously characterized loss of function variant (p.Arg84His) from a 46,XY DSD patient as a positive control (Robevska et al., 2018). We found that like the NR5A1 p.Arg92Trp variant, the p.Ala260Val variant also results in decreased ability to transactivate this male pathway promoter (Figure 3a). This loss of activation was also evident in the presence of the SOX9‐inhibiting factor Forkhead Box L2 (FOXL2), indicating that the variant NR5A1 proteins do not affect the FOXL2‐mediated repression of SOX9 (Figure 3a).

### 3.5 NR5A1 variants alter ovarian pathway activation via reduced WNT signaling activity

As the above assay does not explain why the male pathway is activated and ovarian pathway repressed in these 46,XX (ovo)testicular patients, we turned to female pathways. Previous functional assays on the NR5A1 p.Arg92Trp variant show disruption to both ovarian and testicular pathways (Bashamboo et al., 2016; Igarashi et al., 2017). *NR0B1* is a key gene involved in repression of the testis pathway; its dysregulation may explain these XX phenotypes. In ovarian development, NR5A1 transactivation of SOX9 is repressed by NR0B1 (Igarashi et al., 2017; Ludbrook et al., 2012) proposed that mutant NR5A1 is less responsive to NR0B1. We assessed this by co‐transfecting HEK 293‐T cells with NR0B1, SOX9, and NR5A1 constructs as well as the *mTESCO Sox9* enhancer (Figure 3b). In the presence of wild‐type NR5A1 and SOX9, addition of NR0B1 resulted in dosage‐dependent repression of *Sox9 mTESCO*. Similarly, using both mutant forms of NR5A1, *Sox9 mTESCO* was also repressed in a NR0B1‐dosage‐dependent manner (Figure 3b), although the initial activation of the reporter was lower for both variants as seen in Figure 3a. Thus, these variants are still responsive to NR0B1‐mediated repression of *SOX9*.

Bashamboo et al. (2016) also investigated NR0B1 dysregulation. In the developing gonad, the NR5A1/β‐catenin complex upregulates several targets such as DSS‐AHC critical region on the X chromosome protein 1 (DAX1; encoded by *NR0B1*) (Mizusaki et al., 2003). It has been suggested that mutant NR5A1 causes dysregulation of *NR0B1* and thus loss of SOX9 repression in XX gonads (Bashamboo et al., 2016). We assessed the effect of mutant NR5A1 on NR0B1‐mediated repression of SOX9. To assay any direct effect of these variants on the *NR0B1* promoter, we used a reporter construct containing the upstream *NR0B1* promoter region (994 bp). HEK 293‐T cells were co‐transfected with this *NR0B1* reporter and constructs for wild‐type and variant NR5A1 (Figure 3c). Wild‐type NR5A1 upregulated *NR0B1* promoter activity and both NR5A1 variants showed similar upregulation of the NR0B1 promoter, consistent with the above assay showing that the variants do not change NR5A1‐mediated NR0B1 repression of *SOX9*. To test the NR5A1/β‐catenin complex in NR0B1 upregulation, we introduced β‐catenin. β‐catenin and wild‐type NR5A1 showed a fivefold increase in NR0B1‐reporter activity compared the empty vector controls, but the patient variants significantly repressed this activity (Figure 3d). To further test whether the NR5A1 variants are repressing the WNT/β‐catenin pathway, we looked at the effect of wild‐type and variant NR5A1 on canonical WNT activity using the TOPFlash reporter system (Figure 3e). The TOPFlash reporter shows a 20‐fold induction upon the transfection of the wild‐type β‐catenin construct. Introduction of the wild‐type NR5A1 repressed WNT signaling activity induced by β‐catenin, and we found that both variant forms of NR5A1 repressed this further (*P* < 0.0001). As both variants present in the patient are heterozygous, we also transfected each variant with an equal amount of wild‐type construct. Increased repression of WNT signaling was still observed when each variant NR5A1 was transfected with or without the wild‐type form, indicating some dominant negative effect of the variant allele on the WT. Taken together, these results indicate that variants in *NR5A1* show no reduced NR0B1‐dependent repression of *SOX9* expression or changes in *NR0B1* promoter activation, yet these variants have increased repression of the ovarian‐specific WNT signaling pathway. This may underlie testis development in an XX background.

### 3.6 Additional genomic variants may contribute to oligogenic inheritance

Given the wide phenotypic variation observed in individuals with *NR5A1* variants, recent reports hypothesize that oligogenic inheritance is likely to be at play (Camats, Fernandez‐Cancio, Audi, Schaller, & Fluck, 2018; Robevska et al., 2018). We filtered our massively parallel sequencing data with a list of 116 *NR5A1*‐related genes to identify variants that may act additively with the *NR5A1* variants in these four patients. Sixteen *NR5A1*‐associated variants were identified and summarized in Supporting Information Table S2. In Patient 1, variants were found in *CREBBP*, *GDF9*, *HSD3B1*, *STAR*, and *TG*. Patient 2 had variants in *AR*, *DACH1*, and *ZFPM2*. Patient 3 had two *NR5A1*‐related variants; these were in the *FRAS1* and *MTSS1* genes. Patient 4 had variants in *BMP15*, *MSX2*, *PGR*, *POR*, *PTCH1*, and *RARA*.

## 4 DISCUSSION

During gonadal differentiation, NR5A1 is involved in both activation and repression of the testis pathway. Variants in the *NR5A1* gene can cause a wide variety of DSDs including 46,XY gonadal dysgenesis, 46,XX premature ovarian insufficiency, and recently a single variant (p.Arg92Trp) has been implicated in 46,XX (ovo)testicular DSD (Baetens et al., 2017; Bashamboo et al., 2016; Domenice et al., 2016; Igarashi et al., 2017; Takasawa et al., 2017). The exact mechanism by which this variant activates testis development in a 46,XX individual remains elusive. Here, we have described three patients with 46,XX (ovo)testicular DSD carrying this variant, and in addition, describe a patient with 46,XX ovotesticular DSD with a novel *NR5A1* variant, p.Ala260Val.

As the gonads first begin to differentiate, it is the presence of the Y‐linked *SRY* gene that activates the male genetic pathway, driving differentiation of the testicular Sertoli cells. However, we know that activation of the testis pathway by *SRY* can be bypassed if SOX9, the factor immediately downstream of *SRY*, is upregulated (Huang, Wang, Ning, Lamb, & Bartley, 1999; Vidal, Chaboissier, de Rooij, & Schedl, 2001). NR5A1 is a known regulator of *SOX9*, thus aberrant activation of the male pathway by NR5A1 p.Arg92Trp was investigated as a cause of sex reversal. Previous functional analyses suggest that NR5A1 p.Arg92Trp cannot activate testicular development via male pathway genes including *Sox9*, *Amh*, and *Cyp11a1* (Bashamboo et al., 2016). Instead these variants lose male‐pathway regulatory ability, this is consistent with the fact that these variants have also been described in 46,XY DSD (Bashamboo et al., 2016; Chan et al., 2015) where activation of testis differentiation is lost and ovarian development takes over. Consistent with these results, we found that neither the p.Arg92Trp variant nor the novel p.Ala260Val variant had increased *SOX9* activation, thus *in vitro* there is no evidence for *NR5A1* variants abnormally activating the testis pathway.

When no Y‐chromosome is present, the male pathway is not activated, allowing a female specific pathway to drive ovarian differentiation, and repress testis development. The *NR0B1* gene is involved in repression of the testis pathway and its dysregulation may underlie the sex reversal reported in these cases. NR5A1 transactivates *SOX9*, and in ovarian development, this is antagonized by NR0B1 (Ludbrook et al., 2012). Igarashi et al. (2017) proposed that the NR5A1 p.Arg92Trp mutant is unresponsive to NR0B1, resulting in a loss of *SOX9* repression in 46,XX (ovo)testicular DSD cases. By contrast, we showed that both forms of mutant NR5A1 are still responsive to NR0B1 and still allow repression of *SOX9* activity, suggesting that this anti‐testis interaction is maintained in the 46,XX DSD patients. These contradictory results may be in part due to different cell types being used, performing this assay in the native (human fetal ovary) cells would be necessary to establish whether NR0B1 de‐repression is truly playing a role in these phenotypes. Bashamboo et al. (2016) proposed an alternate mechanism whereby the NR5A1 p.Arg92Trp variant has less ability to upregulate the anti‐testis *NR0B1*, perhaps via a loss of synergy with β‐catenin. Ultimately, this would mean that testis factors, such as *SOX9*, are no longer suppressed. We found that both NR5A1 variants showed similar activation of the *NR0B1* promoter. However, we did find that compared to wild‐type, the two variants repressed the β‐catenin‐mediated activation of this promoter construct, indicating a repression of WNT signaling might be at play. In ovarian differentiation, NR5A1 is also necessary for induction of female pathway genes *Wnt4* and *Rspo1* (Combes et al., 2010). Bashamboo et al. (2016) previously found that the NR5A1 p.Arg92Trp mutant has reduced synergy with β‐catenin and loses WNT signaling activation compared to wild‐type NR5A1. We have found that *in vitro* wild‐type NR5A1 represses β‐catenin‐mediated WNT signaling, and that this repression was significantly enhanced by both patient variant NR5A1 forms. This variation in wild‐type NR5A1 activity (Figure 3e vs. Bashamboo et al., 2016) suggests that the NR5A1/β‐catenin complex has both repressive and activating effects on WNT signaling, these could be influenced by factors such as subtle changes in gene dosage or environment. Being able to investigate these interactions in more standardized cellular assays or cell types more closely resembling human embryonic gonad may help to increase reproducibility of such findings in future. Overall, our findings highlight that increased repression of WNT/β‐catenin signaling resulting in reduced NR0B1, and thus reduced testis pathway repression, may underlie the inhibition of ovarian development and the formation of testis tissue in these 46,XX patients.

Given the large number of known target genes for NR5A1, there are likely to be other signaling pathways not investigated here that are affected in these patients, however the inaccessibility of gonadal tissue makes this difficult to assess. Studying additional individuals with these variants may improve our understanding of possible mechanisms. Of interest, a second heterozygous variant at codon 92 with a different amino acid change (p.Arg92Gln) has been found in 46,XX individuals with adrenal insufficiency with or without ovotesticular DSD (Guran et al., 2016; Swartz et al., 2017). The NR5A1 p.Arg92Gln variant shows partial loss of activation of NR5A1 target gene promoters (including *Amh*, *Cyp11a1*, *Cyp19a1*, and *Insl3*; Lin et al., 2007) but does not show loss of synergy with β‐catenin and reduced WNT signaling like the p.Arg92Trp variant does (Bashamboo et al., 2016). Also, the presence of adrenal phenotypes associated with the p.Arg92Gln variant only suggests that this residue change impacts different target genes to the p.Arg92Trp variant, however long term follow up of cases are necessary as adrenal insufficiency may not present until later in life. This highlights how sequence variation at the A‐box motif in NR5A1 can impact binding specificity to target genes; DNA binding studies comparing these mutant proteins with wild‐type NR5A1 could confirm this.

46,XX individuals with the NR5A1 p.Arg92Trp variant cover a wide phenotypic spectrum, for example, gonadal phenotypes include normal ovary, streak gonad, and testis (Baetens et al., 2017; Bashamboo et al., 2016; Igarashi et al., 2017). Incomplete penetrance and variable expressivity are well established features of *NR5A1*‐associated disorders, this is because adreno‐gonadal development is exquisitely sensitive to changes in *NR5A1* gene dosage (Val, Martinez‐Barbera, & Swain, 2007) and may be subject to genetic or environmental modifiers. A recent paper reported a 46,XX sibling pair each with the heterozygous NR5A1 p.Arg92Trp variant displaying markedly different phenotypes, which indicates that environment is an important modifier (Takasawa et al., 2017). Furthermore, these variants are absent or at low frequency in large population databases (e.g., gnomAD) and individuals with the NR5A1 p.Arg92Trp variant have diverse ethnic backgrounds, including African, Hispanic, European, and Asian, suggesting that the variable phenotypic expressivity observed is not a product of founder effect or genetic background. Additional genomic variants may act as modifiers of expression and thus explain some of the variable expressivity observed. A recent report on 46,XY DSDs with *NR5A1* variants explored oligogenic inheritance by filtering exome sequencing variants with a list of *NR5A1*‐associated genes (Camats et al., 2018). Using a similar, albeit targeted, approach, we identified an additional 2–6 variants per patient that may modify *NR5A1* expression or act additively with *NR5A1* to generate the wide phenotypic variation observed (Supporting Information Table S2). For example, Patient 4 had a heterozygous variant in *Cytochrome P450 Oxidoreductase* (*POR*; MIM# 613571) in addition to the NR5A1 p.Ala260Val variant. Variants in this gene are associated with genital anomalies, and combined *POR* (NM_000941.2:c.1370 G>A; p.Arg457His) and NR5A1 (p.Arg92Trp) variants have been reported in a case of 46,XX testicular DSD before (Igarashi et al., 2017). Given that *NR5A1* and *POR* are both involved in steroidogenesis, these two variants may have an additive effect on steroidogenic function; this may apply to other variants we report in known steroidogenic genes including *STAR*, *PTCH1*, and *HSD3B1*. Functional assessment would be required to confirm their pathogenicity and interaction with NR5A1 in these phenotypes. In future, exome sequencing on these and additional individuals (affected and unaffected) with *NR5A1* variants would enable a genome‐wide and unbiased approach to investigating oligogenic inheritance.

In our cohort of patients with *SRY*‐negative 46,XX (ovo)testicular DSD, *NR5A1* variants contribute to 15% (4/26) of cases. This is comparable to *SOX9* enhancer duplications, which underlie 19% (5/26) of cases in this cohort (Croft et al., 2018; Ohnesorg et al., 2017). Consequently, *NR5A1* gene variants should be considered an important cause underlying cases of *SRY*‐negative 46,XX (ovo)testicular DSDs. Screening for *NR5A1* gene variants should be included in routine genetic testing for these patients.

## Supporting information

Supporting Information

## References

[humu23672-bib-0001] Baetens, D , Stoop, H. , Peelman, F. , Todeschini, A.‐L. , Rosseel, T. , Coppieters, F. , … Cools, M. (2017). NR5A1 is a novel disease gene for 46,XX testicular and ovotesticular disorders of sex development. Genetics in Medicine: Official Journal of the American College of Medical Genetics, 19(4), 367–376. 10.1038/gim.2016.118 27490115 PMC5392598

[humu23672-bib-0002] Bashamboo, A. , Donohoue, P. A. , Vilain, E. , Rojo, S. , Calvel, P. , Seneviratne, S. N. , … Achermann, J. C. (2016). A recurrent p.Arg92Trp variant in steroidogenic factor‐1 (NR5A1) can act as a molecular switch in human sex development. Human Molecular Genetics, 25(16), 3446–3453. 10.1093/hmg/ddw186 27378692 PMC5179941

[humu23672-bib-0003] Bashamboo, A. , Eozenou, C. , Jorgensen, A. , Bignon‐Topalovic, J. , Siffroi, J. P. , Hyon, C. , … McElreavey, K. (2018). Loss of Function of the Nuclear Receptor NR2F2, Encoding COUP‐TF2, Causes Testis Development and Cardiac Defects in 46,XX Children. American Journal of Human Genetics, 102(3), 487–493. 10.1016/j.ajhg.2018.01.021 29478779 PMC5985285

[humu23672-bib-0004] Camats, N. , Fernandez‐Cancio, M. , Audi, L. , Schaller, A. , & Fluck, C. E. (2018). Broad phenotypes in heterozygous NR5A1 46,XY patients with a disorder of sex development: An oligogenic origin? European Journal of Human Genetics, 26(9), 1329–1338. 10.1038/s41431-018-0202-7 29891883 PMC6117353

[humu23672-bib-0005] Chan, A. O. , But, W. M. , Lee, C. Y. , Lam, Y. Y. , Ng, K. L. , Loung, P. Y. , … Tse, W. Y. (2015). Aetiological bases of 46,XY disorders of sex development in the Hong Kong Chinese population. Hong Kong Medical Journal, 21(6), 499–510. 10.12809/hkmj144402 26492835

[humu23672-bib-0006] Combes, A. N. , Spiller, C. M. , Harley, V. R. , Sinclair, A. H. , Dunwoodie, S. L. , Wilhelm, D. , & Koopman, P. (2010). Gonadal defects in Cited2‐mutant mice indicate a role for SF1 in both testis and ovary differentiation. The International Journal of Developmental Biology, 54(4), 683–689. 10.1387/ijdb.092920ac 19757380

[humu23672-bib-0007] Croft, B. , Ohnesorg, T. , & Sinclair, A. H. (2018). The role of copy number variants in disorders of sex development. Sexual Development, 12(1‐3), 19–29. 10.1159/000481896 29145200

[humu23672-bib-0008] Doghman, M. , Figueiredo, B. C. , Volante, M. , Papotti, M. , & Lalli, E. (2013). Integrative analysis of SF‐1 transcription factor dosage impact on genome‐wide binding and gene expression regulation. Nucleic Acids Research, 41(19), 8896–8907. 10.1093/nar/gkt658 23907384 PMC3799431

[humu23672-bib-0009] Domenice, S. , Machado, A. Z. , Ferreira, F. M. , Ferraz‐de‐Souza, B. , Lerario, A. M. , Lin, L. , … Mendonca, B. B. (2016). Wide spectrum of NR5A1‐related phenotypes in 46,XY and 46,XX individuals. Birth Defects Research. Part C, Embryo Today, 108(4), 309–320. 10.1002/bdrc.21145 28033660 PMC5347970

[humu23672-bib-0010] Eggers, S. , Sadedin, S. , van den Bergen, J. A. , Robevska, G. , Ohnesorg, T. , Hewitt, J. , … Sinclair, A. H. (2016). Disorders of sex development: Insights from targeted gene sequencing of a large international patient cohort. Genome Biology, 17(1), 243 10.1186/s13059-016-1105-y 27899157 PMC5126855

[humu23672-bib-0011] Ferraz‐de‐Souza, B. , Lin, L. , & Achermann, J. C. (2011). Steroidogenic factor‐1 (SF‐1, NR5A1) and human disease. Molecular and Cellular Endocrinology, 336(1‐2), 198–205. 10.1016/j.mce.2010.11.006 21078366 PMC3057017

[humu23672-bib-0012] Guran, T. , Buonocore, F. , Saka, N. , Ozbek, M. N. , Aycan, Z. , Bereket, A. , … Achermann, J. C. (2016). Rare causes of primary adrenal insufficiency: Genetic and clinical characterization of a large nationwide cohort. Journal of Clinical Endocrinology and Metabolism, 101(1), 284–292. 10.1210/jc.2015-3250 26523528 PMC4701852

[humu23672-bib-0013] Huang, B. , Wang, S. , Ning, Y. , Lamb, A. N. , & Bartley, J. (1999). Autosomal XX sex reversal caused by duplication of SOX9. American Journal of Medical Genetics, 87(4), 349–353. 10588843 10.1002/(sici)1096-8628(19991203)87:4<349::aid-ajmg13>3.0.co;2-n

[humu23672-bib-0014] Igarashi, M. , Takasawa, K. , Hakoda, A. , Kanno, J. , Takada, S. , Miyado, M. , … Fukami, M. (2017). Identical NR5A1 missense mutations in two unrelated 46,XX individuals with testicular tissues. Human Mutation, 38(1), 39–42. 10.1002/humu.23116 27610946

[humu23672-bib-0015] Knarston, I. , Ayers, K. , & Sinclair, A. (2016). Molecular mechanisms associated with 46,XX disorders of sex development. Clinical Science, 130(6), 421–432. 10.1042/CS20150579 26846580

[humu23672-bib-0016] Kohler, B. , Lin, L. , Ferraz‐de‐Souza, B. , Wieacker, P. , Heidemann, P. , Schroder, V. , … Achermann, J. C. (2008). Five novel mutations in steroidogenic factor 1 (SF1, NR5A1) in 46,XY patients with severe underandrogenization but without adrenal insufficiency. Human Mutation, 29(1), 59–64. 10.1002/humu.20588 17694559 PMC2359628

[humu23672-bib-0017] Lin, L. , & Achermann, J. C. (2008). Steroidogenic factor‐1 (SF‐1, Ad4BP, NR5A1) and disorders of testis development. Sexual Development, 2(4‐5), 200–209. 10.1159/000152036 18987494 PMC2645687

[humu23672-bib-0018] Lin, L. , Philibert, P. , Ferraz‐de‐Souza, B. , Kelberman, D. , Homfray, T. , Albanese, A. , … Achermann, J. C. (2007). Heterozygous missense mutations in steroidogenic factor 1 (SF1/Ad4BP, NR5A1) are associated with 46,XY disorders of sex development with normal adrenal function. Journal of Clinical Endocrinology and Metabolism, 92(3), 991–999. 10.1210/jc.2006-1672 17200175 PMC1872053

[humu23672-bib-0019] Ludbrook, L. M. , Bernard, P. , & Bagheri‐Fam, S. (2012). Excess DAX1 leads to XY ovotesticular disorder of sex development (DSD) in mice by inhibiting steroidogenic factor‐1 (SF1) activation of the testis enhancer of SRY‐box‐9 (Sox9). Endocrinology, 153(4), 1948–1958. 22294746 10.1210/en.2011-1428

[humu23672-bib-0020] McElreavey, K. , Vilain, E. , & Abbas, N. (1993). A regulatory cascade hypothesis for mammalian sex determination: SRY represses a negative regulator of male development. Proceedings of the National Academy of Sciences of the United States of America, 90(8), 3368–3372. Retrieved from http://www.pnas.org/content/90/8/3368.short 8475082 10.1073/pnas.90.8.3368 PMC46301

[humu23672-bib-0021] Mizusaki, H. , Kawabe, K. , Mukai, T. , Ariyoshi, E. , Kasahara, M. , Yoshioka, H. , … Morohashi, K.‐i. (2003). Dax‐1 (dosage‐sensitive sex reversal‐adrenal hypoplasia congenita critical region on the X chromosome, gene 1) gene transcription is regulated by wnt4 in the female developing gonad. Molecular Endocrinology, 17(4), 507–519. 10.1210/me.2002-0362 12554773

[humu23672-bib-0022] Morohashi, K. I. , & Omura, T. (1996). Ad4BP/SF‐1, a transcription factor essential for the transcription of steroidogenic cytochrome P450 genes and for the establishment of the reproductive function. The FASEB Journal, 10(14), 1569–1577. 9002548 10.1096/fasebj.10.14.9002548

[humu23672-bib-0023] Ohnesorg, T. , van den Bergen, J. A. , Belluoccio, D. , Shankara‐Narayana, N. , Kean, A. M. , Vasilaras, A. , … Sinclair, A. H. (2017). A duplication in a patient with 46,XX ovo‐testicular disorder of sex development refines the SOX9testis‐specific regulatory region to 24 kb. Clinical Genetics, 372(8), 525 10.1111/cge.12976 28317102

[humu23672-bib-0024] Parker, K. L. , & Schimmer, B. P. (1997). Steroidogenic factor 1: A key determinant of endocrine development and function. Endocrine Reviews, 18(3), 361–377. 10.1210/edrv.18.3.0301 9183568

[humu23672-bib-0025] Parma, P. , Radi, O. , Vidal, V. , Chaboissier, M.‐C. , Dellambra, E. , Valentini, S. , … Camerino, G. (2006). R‐spondin1 is essential in sex determination, skin differentiation and malignancy. Nature Genetics, 38(11), 1304–1309. 10.1038/ng1907 17041600

[humu23672-bib-0026] Robevska, G. , van den Bergen, J. A. , Ohnesorg, T. , Eggers, S. , Hanna, C. , Hersmus, R. , … Sinclair, A. H. (2018). Functional characterization of novel NR5A1 variants reveals multiple complex roles in disorders of sex development. Human Mutation, 39(1), 124–139. 10.1002/humu.23354 29027299 PMC5765430

[humu23672-bib-0027] Ropke, A. , Tewes, A. C. , Gromoll, J. , Kliesch, S. , Wieacker, P. , & Tuttelmann, F. (2013). Comprehensive sequence analysis of the NR5A1 gene encoding steroidogenic factor 1 in a large group of infertile males. European Journal of Human Genetics, 21(9), 1012–1015. 10.1038/ejhg.2012.290 23299922 PMC3746266

[humu23672-bib-0028] Roy, A. , Kucukural, A. , & Zhang, Y. (2010). I‐TASSER: A unified platform for automated protein structure and function prediction. Nature Protocols, 5(4), 725–738. 10.1038/nprot.2010.5 20360767 PMC2849174

[humu23672-bib-0029] Swartz, J. M. , Ciarlo, R. , Guo, M. H. , Abrha, A. , Weaver, B. , Diamond, D. A. , … Hirschhorn, J. N. (2017). A 46,XX ovotesticular disorder of sex development likely caused by a steroidogenic factor‐1 (NR5A1) variant. Hormone Research in Paediatrics, 87(3), 191–195. 10.1159/000452888 27855412 PMC5388569

[humu23672-bib-0030] Takasawa, K. , Igarashi, M. , Ono, M. , Takemoto, A. , Takada, S. , Yamataka, A. , … Kashimada, K. (2017). Phenotypic variation in 46,XX disorders of sex development due to the NR5A1 p.R92W variant: A sibling case report and literature review. Sexual Development, 11(5‐6), 284–288. 10.1159/000485868 29393271

[humu23672-bib-0031] Tomaselli, S. , Megiorni, F. , De Bernardo, C. , Felici, A. , Marrocco, G. , Maggiulli, G. , … Grammatico, P. (2008). Syndromic true hermaphroditism due to an R‐spondin1 (RSPO1) homozygous mutation. Human Mutation, 29(2), 220–226. 10.1002/humu.20665 18085567

[humu23672-bib-0032] Val, P. , Lefrancois‐Martinez, A. M. , Veyssiere, G. , & Martinez, A. (2003). SF‐1 a key player in the development and differentiation of steroidogenic tissues. Nuclear Receptor, 1(1), 8 10.1186/1478-1336-1-8 14594453 PMC240021

[humu23672-bib-0033] Val, P. , Martinez‐Barbera, J. P. , & Swain, A. (2007). Adrenal development is initiated by Cited2 and Wt1 through modulation of Sf‐1 dosage. Development, 134(12), 2349–2358. 10.1242/dev.004390 17537799

[humu23672-bib-0034] Venselaar, H. , Beek Te , T. A. , Kuipers, R. K. , Hekkelman, M. L. , & Vriend, G. (2010). Protein structure analysis of mutations causing inheritable diseases. An e‐Science approach with life scientist friendly interfaces. BMC Bioinformatics, 11, 548 10.1186/1471-2105-11-548 21059217 PMC2992548

[humu23672-bib-0035] Vidal, V. P. , Chaboissier, M. C. , de Rooij, D. G. , & Schedl, A. (2001). Sox9 induces testis development in XX transgenic mice. Nature Genetics, 28(3), 216–217. 10.1038/90046 11431689

[humu23672-bib-0036] Vilain, E. (2011). The genetics of ovotesticular disorders of sex development. Advances in Experimental Medicine and Biology, 707, 105–106. 21691964 10.1007/978-1-4419-8002-1_22

[humu23672-bib-0037] Voican, A. , Bachelot, A. , Bouligand, J. , Francou, B. , Dulon, J. , Lombes, M. , … Guiochon‐Mantel, A. (2013). NR5A1 (SF‐1) mutations are not a major cause of primary ovarian insufficiency. Journal of Clinical Endocrinology and Metabolism, 98(5), E1017–E1021. 10.1210/jc.2012-4111 23543655

[humu23672-bib-0038] Zhang, Y. (2008). I‐TASSER server for protein 3D structure prediction. BMC Bioinformatics, 9, 40 10.1186/1471-2105-9-40 18215316 PMC2245901

