# Supplementary material for: NR5A1 gene variants repress the ovarian‐specific WNT signaling pathway in 46,XX disorders of sex development patients
Source: Hum Mutat. 2018 Nov 30;40(2):207–16. doi: 10.1002/humu.23672 (PMC6492147; doi:10.1002/humu.23672)
Supplement: Supplementary file 1 — Supporting Information [file HUMU-40-207-s001.pdf]

Supplementary Table S1. Mutagenesis and Cloning Primers.

Name	Tm	GC%	Secondary structure	Primer Dimer	Base count	Sequence (5' -> 3')	Species	Experiment
NR5A1_c.C274T_ex4_For	81.2	58.1	Very weak	No	31	GCCCAAACTTGTTCCAGCCACCCCTCATACG	Human	Site-directed mutagenesis
NR5A1_c.C274T_ex4_Rev	81.2	58.1	Very weak	No	31	CGTATGAGGGGTGGCTGGAACAAGTTTGGGC	Human	Site-directed mutagenesis
NR5A1_c.C779T_p.A260V_For	84.1	78.3	Very strong	No	23	GGCCGAAGGCCACCGGCTGGTCG	Human	Site-directed mutagenesis
NR5A1_c.C779T_p.A260V_Rev	84.1	78.3	Very strong	Yes	23	CGACCAGCCGGTGGCCTTCGGCC	Human	Site-directed mutagenesis
hDAX1_XhoI_For	59.2	43.5	None	No	23	TTTCTCGAGCCTATTGGATACTATTACCTGGG	Human	Cloning, luciferase assay
hDAX1_HindIII_Rev	60.9	50	Moderate	No	20	TTTAAGCTTGCATGTTGTAGAGGATGCTG	Human	Cloning, luciferase assay

Supplementary Table S2. Additional genomic variants identified in 46,XX (ovo)testicular DSD cases.

Patient ID	Gene	Chr	DNA change	Protein change	Consequence	Zygosity	Inheritance	In silico predictions	dbSNP ID	ClinVar	gnomAD frequency
1	*CREBBP*	16	NM_001079846.1:c.1537C>A	p.Leu513Ile	missense	Heterozygous	N/A	3/4 damaging	rs61753381	Conflicting interpretations of pathogenicity. Reported in: Rubinstein-Taybi syndrome.	0.009668

1	*GDF9*	5	NM_005260.5:c.307C>T	p.Pro103Ser	missense	Heterozygous	N/A	3/4 damaging	rs61754583	Not found	0.002925
1	*HSD3B1*	1	NM_000862.2:c.674A>G	p.Tyr225Cys	missense	Heterozygous	N/A	3/4 damaging	rs774738158	Not found	0.0001662
1	*STAR*	8	NM_000349.2:c.820C>T	p.Arg274Cys	missense	Heterozygous	N/A	3/4 damaging	rs138161253	Not found	6.37E-05
1	*TG*	8	NM_003235.4:c.455G>A	p.Arg152His	missense	Heterozygous	N/A	3/4 damaging	rs114781869	Not found	0.0007271
2	*AR*	X	NM_000044.4:c.1174C>T	p.Pro392Ser	missense	Heterozygous	N/A	3/4 damaging	rs201934623	Conflicting interpretations of pathogenicity. Reported in: Hypospadias, Partial androgen insensitivity syndrome.	0.004138
2	*DACH1*	13	NM_004392.6:c.345C>A	p.Asn115Lys	missense	Heterozygous	N/A	0/4 benign	rs1264546940	N/A	N/A

2	*ZFPM2*	8	NM_012082.3:c.292G>A	p.Asp98Asn	missense	Heterozygous	N/A	2/4 damaging	rs202217256	Benign. Reported in: 46,XY sex reversal 9.	0.002608
3	*FRAS1*	4	NM_025074.6:c.9806G>A	p.Arg3269Gln	missense	Heterozygous	Maternal	3/4 damaging	rs61729366	Conflicting interpretations of pathogenicity, risk factor. Reported in: Cryptophthalmos syndrome, Congenital diaphragmatic hernia.	0.005271
3	*MTSS1*	8	NM_014751.4:c.1187T>C	p.Ile396Thr	missense	Heterozygous	Paternal	1/4 damaging	rs548792952	Not found	0.00124
4	*BMP15*	X	NM_005448.2:c.782_783delinsTCT	p.Ser261delinsSerLeu	inframe insertion	Heterozygous	N/A	N/A	rs111889793	Not found	N/A
4	*MSX2*	5	NM_002449.4:c.95A>T	p.Glu32Val	missense	Heterozygous	N/A	0/4 benign	rs780593593	Not found	2.57E-05
4	*PGR*	11	NM_000926.4:c.662T>A	p.Val221Asp	missense	Heterozygous	N/A	3/4 damaging	rs200322178	Not found	0.000291

4	*POR*	7	NM_000941.2:c.1709G>A	p.Arg570His	missense	Heterozygous	N/A	4/4 damaging	rs372955296	Not found	0.0001788
4	*PTCH1*	9	NM_000264.4:c.1612G>C	p.Gly538Arg	missense	Heterozygous	N/A	3/4 damaging	rs1347882326	N/A	N/A
4	*RARA*	17	NM_000964.3:c.128C>G	p.Thr43Ser	missense	Heterozygous	N/A	0/4 benign	rs116538651	Not found	0.001987

Footnote: In silico (only available for missense variants) predictors: Mutation Taster, PolyPhen2, SIFT, LRT; damaging: deleterious, probably deleterious or possibly deleterious.

Supplementary Table S3. Numerical output from the in silico analyses of the missense changes identified in *NR5A1*.

Gene	DNA change	Protein change	SIFT score	SIFT Pred	Polyphen2 score	Polyphen2 Pred	LRT score	LRT Pred	Mutation Taster score	Mutation Taster Pred	CADD Phredlike score
NR5A1	c.274C>T	p.Arg92Trp	1	D	1.000	P	1	D	0.999884	Di	29.3
NR5A1	c.779C>T	p.Ala260Val	0.98	D	0.695	P	0.999994	D	0.998770	Po	22.2

Footnote: D, Deleterious; Di, Disease causing; P, Possibly deleterious; Po, Polymorphism; Pred, Prediction.
